# Molecular investigation of vector-borne parasitic infections in dogs in Northeast India

**DOI:** 10.1186/s13071-019-3389-8

**Published:** 2019-03-26

**Authors:** Kalyan Sarma, Yaarit Nachum-Biala, Mritunjay Kumar, Gad Baneth

**Affiliations:** 1 Department of Veterinary Medicine, College of Veterinary Sciences and Animal Husbandry, Central Agricultural University, Selesih, Aizawl, Mizoram India; 2 Koret School of Veterinary Medicine, The Hebrew University of Jerusalem, P.O. Box 12, Rehovot, 76100 Israel; 3 Department of Teaching Veterinary Clinical Complex, College of Veterinary Sciences and Animal Husbandry, R. K. Nagar, Tripura (W) 799008 India

**Keywords:** *Babesia gibsoni*, *Hepatozoon canis*, *Anaplasma platys*, *Dirofilaria immitis*, *Acanthocheilonema reconditum*, Mizoram, Tripura, Northeast India

## Abstract

**Background:**

Information on the status of vector-borne pathogens among canines in Northeast India is lacking, particularly for the states of Mizoram and Tripura close to the Myanmar border. Blood samples collected from 130 dogs, 80 from Mizoram and 50 from Tripura, were examined in this study.

**Methods:**

Polymerase chain reaction (PCR) was performed for filariid worms, *Babesia*, *Hepatozoon*, *Ehrlichia* and *Anaplasma* spp. and DNA sequencing was then carried out to identify pathogens at the species level.

**Results:**

Vector-borne pathogens were detected in 52% (68/130) of the sampled dogs. The most prevalent pathogen was *Babesia gibsoni* detected in 56/130 (43%) dogs, followed by *Hepatozoon canis* in 50/130 (38%), *Anaplasma platys* in 4/130 (3%), *B. vogeli* in 4/130 (3%), *Acanthochelionema reconditum* in 3/130 (2%) and *Dirofilaria immitis* in 2/130 (2%). Forty-four dogs (34%) were co-infected with two or more pathogens. The most common co-infection observed was with *B. gibsoni* + *H. canis* (34%) followed by triple-infection with *B*. *gibsoni* + *H. canis* + *A. platys* (3%), and *B. gibsoni* + *B. vogeli* + *H. canis* (3%). The infection rate was higher in Mizoram (58%) than in Tripura (44%).

**Conclusions:**

The high prevalence of infection in the studied dog population, especially with *B. gibsoni* and *H. canis*, indicates that vector-borne diseases pose a serious threat to the health of dogs in this area of Northeast India. Prevention of vector-borne diseases by using topical acaricides and heartworm preventative treatment would be of great benefit for reducing the threat of vector-borne diseases in the study area.

## Background

**Fig. 1:**
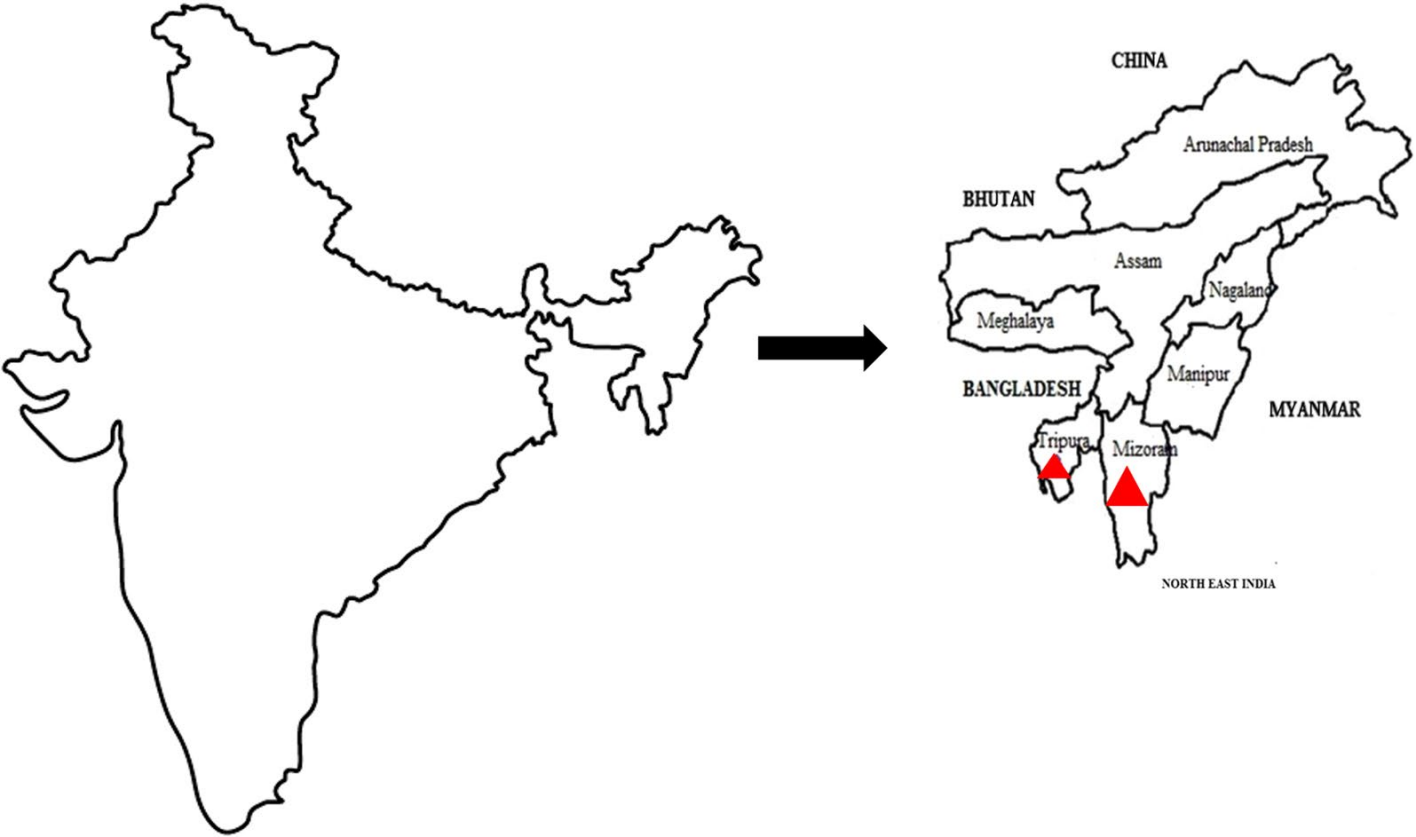
Map of India with enlargement of Northeast India showing the states where samples were collected, as indicated by red triangles

Relatively little is known about the infection of dogs with vector-borne pathogens in some parts of India. The Northeast Region (NER) of India includes eight States: Arunachal Pradesh, Assam, Manipur, Meghalaya, Mizoram, Nagaland, Tripura and Sikkim (Fig. 1). The climate in this area ranges from subtropical to temperate and the terrain is mostly mountainous. The human population density varies from 13 persons/km^2^ in Arunachal Pradesh to 343 persons/km^2^ in Assam. The NER is bordered by China, Myanmar and Bangladesh and there is a possibility of animal and disease transmission from these countries. India’s dog population is estimated at over 25 million and 80% of this population includes either partially restricted community (stray) or feral (unrestricted) dogs [1]. Seventeen percent of Indian households were reported to own a pet or domesticated dog in 2003 [2]. Despite the importance of canine vector-borne diseases (CVBD), molecular-based studies on CVBD prevalence in dogs from different states of the NER are lacking. Such information is a prerequisite for designing appropriate strategies for disease control. The purpose of this study was to evaluate infection with different infectious agents causing CVBD in the states of Mizoram and Tripura located at the southern borders of the NER of India.

## Methods

### Dogs included in the study

The study included 130 dogs of which 75 were privately owned pets, 30 were working dogs and 25 were stray dogs of different breeds. All dogs were more than 2 months-old and their age was either reported by their owners or estimated based on dentition, body size and appearance for non-owned animals. Dogs were divided into three age groups: below 1 year of age, 1–5 years of age and above 5 years-old. These dogs were presented during the year 2016 with different clinical disease conditions at the Teaching Veterinary Clinical Complex, College of Veterinary Sciences and Animal Husbandry, Selesih, Mizoram (*n* = 80; 23.36°N, 92.8°E) and the Teaching Veterinary Clinical Complex, College of Veterinary Sciences and Animal Husbandry, R. K. Nagar, Tripura (*n* = 50; 23.84°N, 91.28°E). The dogs selected were infested with ticks or had a history of tick exposure and their clinical findings included lethargy, dehydration, anorexia, weight loss, fever, lameness, hemorrhages, pale mucous membrane, lymphadenomegaly, gastrointestinal alterations, jaundice, dermatological or ocular abnormalities, and anemia.

### Collection of blood

Two milliliters of blood were collected from each of the dogs in EDTA vials and stored at -20 °C until transported to the Laboratory for Zoonotic and Vector-Borne Diseases at the Koret School of Veterinary Medicine, Hebrew University, Rehovot, Israel.

### DNA extraction

DNA was extracted from 200 μl of the EDTA-buffered whole blood using a commercially available DNA extraction kit (illustra blood genomicPrep Mini Spin Kit, GE Healthcare, Little Chalfont, UK), according to the manufacturer’s instructions. The extracted DNA was eluted in 200 μl of elution buffer and stored at -20 °C until further analysis.

### Real-time PCR for the detection of *Ehrlichia* and *Anaplasma* spp.

The detection of *Ehrlichia* and *Anaplasma* spp. was performed by screening all DNA samples by a real-time PCR assay targeting a 123 bp fragment of the *16S* rRNA gene (E.c 16S-fwd/E.c 16S-rev) [3]. Positive samples were tested by a conventional nested PCR using the ECC and ECB primers targeting a 500 bp fragment of the *16S* rRNA gene in the first round of PCR followed by a second round of PCR using *E. canis*-specific primers (Ecan/HE3) and *A. platys*-specific primers (ApysF/ApysR) [4] (Table 1). DNA extracted from an *E. canis* cell culture (source: Koret School of Veterinary Medicine, Rehovot, Israel) and DNA extracted from a dog infected with *A. platys* confirmed by PCR and sequencing (source: Koret School of Veterinary Medicine, Rehovot, Israel) were used as positive controls.

**Table 1:** Targeted organisms and list of primers used in this study

Target organism	Primer	Sequence (5′–3′)	Fragment length (bp)	Reference
*Babesia* spp. & *Hepatozoon* spp.	Piroplasmid-F	CCAGCAGCCGCGGTAATTCCTTTCGC	400	[6]
	Piroplasmid-R	AGTAGTTYGTCTTTAACAAATCT		
*Babesia* spp.	Babesia18S-F	CCGTGCTAATTGTAGGGCTAATACA	551	[7]
	Babesia18S-R	GCTTGAAACACTCTARTTTTCTCAAAG		
*Babesia* spp.	455-479F^a^	GTCTTGTAATTGGAATGATGGTGAC	340	[8]
	793-772R^b^	ATGCCCCCAACCGTTCCTATTA		
*Babesia gibsoni*	BgibAsia-F^c^	ACTCGGCTACTTGCCTTGTC	185	[8]
*Babesia vogeli*	BCV-F^d^	GTTCGAGTTTGCCATTCGTT	192	[8]
*Hepatozoon* spp.	Hepatozoon18S-F	GGTAATTCTAGAGCTAATACATGAGC	574	[7]
	Hepatozoon18S-R	ACAATAAAGTAAAAAACAYTTCAAAG		
*Ehrlichia* spp.	E.c 16S-fwd	TCGCTATTAGATGAGCCTACGT	123	[3]
*Anaplasma* spp.	E.c 16S-rev	GAGTCTGGACCGTATCTCAGT		
*Anaplasmataceae*	ECB	CGTATTACCGCGGCTGCTGGCA	500	[4]
	ECC	AGAACGAACGCTGGCGGCAAGC		
*Ehrlichia canis*	ECAN5	CAATTATTTATAGCCTCTGGCTATAGGA	400	[4]
	HE3	ATAGGGAAGATAATGACGGTACCTATA		
*Anaplasma platys*	ApysF	GTCGAACGGATTTTTGTCGT	200	[4]
	Apys	RTAGATCACCGCCTTGGTAGG		
Filariid worms	Forward	TTTAAACCGAAAAAATATTGACTGAC	115	[5]
	Reverse	AAAAACTAAACAATCATACATGTGCC		

^a^Nested PCR outer forward primer

^b^Nested PCR outer reverse primer

^c^Nested PCR inner reverse primer

^d^Nested PCR inner reverse primer

Real-time PCR was performed in a total volume of 20 μl containing 4 μl of DNA, 400 nM of each primer, 10 μl of Maxima Hot Start PCR Master Mix (2×) (Thermo Scientific, Epsom, UK), 50 μM SYTO9 solution (Invitrogen, Carlsbad, CA, USA) and sterile DNase/RNase-free water (Biological Industries, Beit Haemek, Israel), using a StepOne-Plus real-time PCR thermal cycler (Applied Biosystems, Foster City, CA, USA). Initial denaturation for 5 min at 95 °C was followed by 40 cycles of denaturation at 95 °C for 5 s, annealing and extension at 59 °C for 30 s, and final extension at 72 °C for 20 s. Amplicons were subsequently subjected to a melt step with the temperature raised to 95 °C for 10 s and then lowered to 60 °C for 1 min. The temperature was then raised to 95 °C at a rate of 0.3 °C/s. Amplification and melt profiles were analyzed using the StepOne-Plus software v.2.2.2 (Applied Biosystems). Negative uninfected dog DNA, and non-template DNA controls were used in each run for all pathogens.

Conventional PCR was performed in a total volume of 25 μl using the PCR-ready High Specificity mix (Syntezza Bioscience, Jerusalem, Israel) with 400 nM of each primers and sterile DNase/RNase-free water (Sigma, St. Louis, MO, USA). Amplification was performed using the Tone 96G programmable conventional thermocycler (Biometra, Gottingen, Germany). Initial denaturation at 95 °C for 5 min, was followed by 35 cycles of denaturation at 95 °C for 30 s, annealing and extension at 65°C for 30 s (for ECC/ECB) and 62 °C for 30 s (for ApysF/ApysR) and 10 cycles of 62 °C for 30 s followed by 25 cycles of 60 °C for 30 s for the ECAN5/HE3 primers, and final extension at 72 °C for 30 s. After the last cycle, the extension step was continued for a further 5 min. PCR products were electrophoresed on 1.5 % agarose gels stained with ethidium bromide and evaluated under UV light for the size of amplified fragments by comparison to a 100 bp DNA molecular weight marker.

### Real-time PCR for the detection of microfilariae

A real-time PCR was performed using primers that target a partial sequence of the mitochondrial *12S* gene of filariids of approximately 115 bp. These were designed to detect *D. immitis*, *Brugia malayi* and *Brugia pahangi* [5] (Table 1), but they are also able to amplify the DNA of other filariid worms. Three microliters of each DNA sample were diluted in a final volume of 20 μl with 10 μl of Maxima Hot Start PCR Master Mix (Thermo Scientific), 4.8 μl of sterile PCR grade water, 0.6 μl of SYTO-9 (Invitrogen) and 400 nM of each primer. The protocol was modified by performing an initial hold of 4 min at 95 °C and 50 cycles of 5 s at 95 °C, 15 s at 58 °C and 10 s at 72 °C. The melt curve was constructed from 65–95 °C with increments of 0.1 °C/s. Reactions were performed with a StepOne-Plus real-time PCR thermal cycler (Applied Biosystems). All runs included a non-template control (NTC) with PCR-grade water and DNA from a laboratory bred pathogen-free dog blood sample. DNA extracted from *Dirofilaria repens*-positive blood samples from Israel were employed as positive controls for the standardization of the assay. All positive amplicons obtained in the study were confirmed by sequencing.

### Conventional PCR assays for *Babesia* and *Hepatozoon* spp

Molecular detection of *Babesia* and *Hepatozoon* species was performed by screening all DNA samples by a conventional PCR assay targeting a 350–400 bp fragment of the *18S* rRNA gene (Piroplasmid-F/Piroplasmid-R [6]). In order to identify cases of co-infection, positive samples were tested by additional PCRs using primers specifically designed for the detection of a fragment of the *18S* rRNA gene of *Babesia* spp. (Babesia18S-F/Babesia18S-R [7]) and *Hepatozoon* spp. (Hepatozoon18S-F/ Hepatozoon18S-R [7]) (Table 1). DNA extracted from a dog infected with *Hepatozoon canis* and a dog infected with *Babesia gibsoni* confirmed by PCR and sequencing were used as positive controls (source: Koret School of Veterinary Medicine, Rehovot, Israel).

Conventional PCR was performed in a total volume of 25 μl using the PCR-ready High Specificity mix (Syntezza Bioscience) with 400 nM of each primers and sterile DNase/RNase-free water (Sigma). Amplification was performed using the Tone 96G programmable conventional thermocycler (Biometra). Initial denaturation at 95 °C for 5 min, was followed by 35 cycles of denaturation at 95 °C for 30 s, annealing and extension at 64 °C for 30 s (for Piroplasmid-F/Piroplasmid-R) or 58 °C for 30 s (for Babesia18S-F/Babesia18S-R) or 50 °C for 30 s (for Hepatozoon18S-F/ Hepatozoon18S-R), and final extension at 72 °C for 30 s. After the last cycle, the extension step was continued for a further 5 min. PCR products were electrophoresed on 1.5 % agarose gels stained with ethidium bromide and evaluated under UV light for the size of amplified fragments by comparison to a 100 bp DNA molecular weight marker.

### Nested PCR for the detection of co-infection with *Babesia gibsoni* and *Babesia vogeli*

Samples which were positive for *Babesia* spp. by conventional PCR and confirmed by sequencing were further tested to rule out co-infection with *B. gibsoni* and *B. vogeli* using a semi-nested PCR protocol [8]. Outer forward primer 455-479F and outer reverse primer 793-772R were used for the first round PCR. For the second round PCR, outer reverse primer was paired with primer BgibAsia-F to detect *B. gibsoni* and with primer BCV-F to specifically detect *B. vogeli* [8]. Amplification was performed using the Tone 96G programmable conventional thermocycler (Biometra). Initial denaturation at 95 °C for 5 min, was followed by 40 cycles of denaturation at 95 °C for 45 s, annealing and extension at 58 °C for 45 s, and final extension at 72 °C for 45 s. After the last cycle, the extension step was continued for a further 5 min. PCR products were electrophoresed on 1.5% agarose gels stained with ethidium bromide and evaluated under UV light for the size of amplified fragments by comparison to a 100 bp DNA molecular weight marker.

### DNA sequencing

All positive PCR products except for the nested PCR amplicons were sequenced using Big-Dye Terminator v.3.1 Cycle Sequencing Kit (Thermo Scientific) and an ABI PRISM 3100 Genetic Analyzer (Applied Biosystems), at the Center for Genomic Technologies, Hebrew University of Jerusalem, Israel. DNA sequences were evaluated with the ChromasPro software v.2.1.1 (Technelysium Pty Ltd., South Brisbane, Australia) and compared for similarity with sequences available in GenBank using the BLAST program (http://www.ncbi.nlm.nih.gov/BLAST/). The species identity found was determined according to the closest BLAST match with an identity of 97–100% to an existing GenBank accession.

## Results

The study included 83 (64%) male and 47 (36%) female dogs (Table 2). Fifty-three dogs (41%) were below 1 year of age, 56 (43%) were 1–5 years-old, and 21 (16%) were above 5 years of age. Sixty-seven (52%) dogs were purebred, 29 (22%) were crosses of purebred dogs and 34 (26%) were local mongrels that could not be associated to any breed. Of the 130 dogs tested, 68 (52%) were found to be infected with CVBD agents: 46 of 80 (58%) dogs in Mizoram and 22 of 50 (44%) in Tripura (Table 3). There was no significant difference in CVBD agent’s prevalence between the study areas (Chi-square test, *χ*^2^ = 2.248, *df* = 1, *P* = 0.134)

**Table 2:** Distribution of infection with CVBD agents according to sex, age and dog breed

Variable	Total no. of dogs	Total no. of CVBD-positive dogs (%)	No. of positive dogs					
			*B. gib*	*H. can*	*D. imm*	*A. rec*	*B. vog*	*A. pla*
Sex								
Male	83	44 (53)	36	30	2	3	1	3
Female	47	24 (51)	20	20	0	0	3	1
Age (years)								
0–1	53	28 (53)	22	17	1	2	1	0
1–5	56	23 (41)	20	20	1	0	2	2
> 5	21	17 (81)	14	13	0	1	1	2
Breed								
Pure	67	38 (57)	8	2	2	2	4	2
Cross	29	12 (41)	2	2	0	1	0	1
Local	34	18 (53)	3	2	0	0	0	1

*Abbreviations*: *B. gib*, *B. gibsoni*; *H. can*, *H. canis*, *D. imm*, *D. immitis*; *A. rec*, *A. reconditum*, *B. vog*, *B. vogeli*; *A. pla*, *A. platys*

**Table 3:** Molecular detection of vector-borne pathogens in dogs from Mizoram and Tripura states in Northeast India

Pathogen	Total no. of dogs infected with each pathogen according to state (%)		Total no. of infected dogs (%)
	Mizoram (*n* = 80)	Tripura (*n* = 50)	
*Babesia gibsoni*	37 (46)	19 (38)	56 (43)
*Babesia vogeli*	4 (5)	–	4 (3)
*Hepatozoon canis*	34 (43)	16 (32)	50 (38)
*Dirofilaria immitis*	2 (3)	–	2 (2)
*Acanthocheilonema reconditum*	3 (4)	–	3 (2)
*Anaplasma platys*	3 (4)	1 (2)	4 (3)
Single infections			
*Babesia gibsoni*	6 (8)	6 (12)	12 (9)
*Babesia vogeli*	1 (1)	–	1 (1)
*Hepatozoon canis*	3 (4)	3 (6)	6 (5)
*Dirofilaria immitis*	2 (3)	–	2 (2)
*Acanthocheilonema reconditum*	3 (4)	–	3 (2)
Co-infections			
*B. gibsoni* + *H. canis*	25 (31)	12 (24)	37 (28)
*B. gibsoni* + *B. vogeli* + *H. canis*	3 (4)	–	3 (2)
*B. gibsoni* + *H. canis* + *A. platys*	3 (4)	1 (2)	4 (3)
Total	46 (58)	22 (44)	68 (52)

The prevalence of CVBD agents according to sex, age and breed of the animal (pure, cross and local) is presented in Table 2. No significant differences were observed between male (44/83, 53%) and female dogs (24/47, 51%) (Chi-square test, *χ*^2^ = 046, *df* = 1, *P* = 0. 831). There was no significant difference in the prevalence of CVBD agents among the different age groups (Chi-square test, *χ*^2^ = 3.059, *df* = 2, *P* = 0.217). There was also no significant difference in CVBD agent’s prevalence between purebred, crossbred and local mongrel dogs (Chi-square test, *χ*^2^ = 0.93, *df* = 2, *P* = 0.761) (Table 2).

The study revealed that *B. gibsoni* infection was the most common pathogen among the 130 studied dogs (56/130, 43%), followed by *H. canis* (50/130, 38%), *B. vogeli* (4/130, 3%) *Anaplasma platys* (4/130, 3%), *Acanthocheilonema reconditum* (3/130, 2%) and *Dirofilaria immitis* (2/130, 2%). Co-infections with *B. gibsoni* and *H. canis* were most prevalent (44/130, 34%) followed by triple-infection with *B. gibsoni* + *H. canis* + *A. platys* (4/130, 3%) and triple-infection with *B. gibsoni* + *B. vogeli* + *H. canis* (4/130, 3%) (Table 3). Of the *Babesia* species identified by DNA sequencing and compared by BLAST analysis to the closest GenBank matches, 56 samples were 100% identical to *B. gibsoni* (GenBank: KY563118.1) from an Indian dog and one was 100% identical to *B. vogeli* (GenBank: MG758132.1) from a tick in Australia. All 50 *H. canis* samples had sequences 98–100% identical to *H. canis* (GenBank: KT267960.1) from a Malaysian dog. The four *A. platys* sequences were 100% identical to *A. platys* (GenBank: KU569704.1) from a wild ungulate in Kenya. The three *A. reconditum* sequences were 99% identical to *A. reconditum* (GenBank: JF461460.1) from an Italian dog, and the two *D. immitis* sequences were 99% identical to *D. immitis* (GenBank: KU885998.1) from a mosquito in Serbia.

## Discussion

The results of this study indicate that CVBD agents are very frequent among dogs suspected of vector-borne infection in the NER of India. Tick-borne protozoan infections with *B. gibsoni* and *H. canis* were more frequent than filarial infections with *D. immitis* and *A. reconditum*. In contrast to the high prevalence of *B. gibsoni* and *H. canis*, tick-borne rickettsial infections with *Anaplasma* spp. and *E. canis* were rarer with no identification of *E. canis* and a relatively small number of *A. platys*-infected dogs. To our knowledge, this study is the first investigation of CVBD agents in the studied region of India using molecular techniques, thus enabling determination of pathogen species which is often not possible using light microscopy.

The findings of the present study provide a very different picture of infection with CVBD agents in the NER from that described for dogs in other parts of India. A study by Abd Rani et al. [9] in which stray and refuge dogs were tested by PCR for tick-borne infections revealed that dogs in the Delhi area were predominantly infected by *E. canis* (40%), *H. canis* (38%) and *B. vogeli* (9%) with no detection of *B. gibsoni*. Dogs from Mumbai were infected with *H. canis* (44%), *E. canis* (27%) and *B. vogeli* (7%), again with no record of *B. gibsoni* [9]. Dogs from Ladakh in northern India were found infected only with *H. canis* (24%), and dogs from Sikkim, which was the closest location to Mizoram and Tripura surveyed in the present study, had a low infection rate with *B. vogeli* (2%) and *B. gibsoni* (1%) [9]. *Babesia gibsoni* was found to be the most prevalent blood-borne pathogen in our study but rare in other parts of northern and central India. It is, however, frequent in dogs in southern India. It was reported to have a prevalence of 47% by PCR among 150 client-owned dogs in Kerala [10] and was detected by microscopy of stained blood smears in 57% of 1986 dogs diagnosed with blood-borne pathogens during a six year study in Chennai, Tamil Nadu, southern India [11]. The latter study reported *E. canis* in 23%, *H. canis* in 11% and *B. canis* in 6% of the dogs infected with vector-borne pathogens [11].

A study based on microscopy of 525 dog blood smears reported from six states in the NER of India, including Mizoram and Tripura, reported that 12% of the dogs were positive for tick-borne pathogens including *E. canis* (5%), *A. platys* (2%), *B. gibsoni* (2%), *Babesia canis* (presumably *B. vogeli*; 1.5%) and *H. canis* (1.5%) [12]. Although these findings are different from those found in the present study, particularly with regard to *E. canis*, the earlier study did not detail the specific states and locations of the positive dogs [12].

While these reports from different parts of India described different populations of dogs and were made by dissimilar detection techniques, thus making a comparison difficult, the proportions between the different infecting agents found and the total absence of some pathogens in certain areas are of value in understanding the distributions of CVBD agents in the Indian sub-continent.

The distribution of tick-borne diseases is related to the presence of their tick vectors. The distribution of tick species in India has been described in several reports but lacks details on specific locations [13]. The only tick species that infests dogs reported in Mizoram is *Rhipicephalus sanguineus* (*s.l.*), while in Tripura tick infestation on dogs has yet to be documented [13]. This could be due to insufficient surveillance rather than the absence of tick infesting dogs in these states. *Hepatozoon canis* is known to be transmitted by *R. sanguineus* (*s.l.*) and also by *Rhipicephalus turanicus* which is reported in other areas of India [13–15]. *Rhipicephalus sanguineus* (*s.l.*) also transmits *B. vogeli* and *E. canis* [16, 17] and there is evidence that it transmits *A. platys* [18]. *Babesia gibsoni* is transmitted by *Haemaphysalis longicornis* [19], *H. hystricis* (recently reported as a vector in Taiwan) and perhaps by *R. sanguineus* [20]. *Haemaphysalis longicornis* has been reported from cattle in Arunachal Pradesh in the NER of India [21] and is therefore possibly also present in Mizoram and Tripura while *H. hystricis* was also reported in other areas of India [13]. It is therefore likely that all tick-borne agents detected in our study in dogs are transmitted locally by tick species recognized as vectors and present in Mizoram and Tripura states.

Filarial infections have been reported in dogs in many parts of India and also differ considerably in their prevalence between regions in the Indian sub-continent [22]. Mosquito vectors of filariid worms are prevalent in large areas of India and are reported in several local studies but there is currently no detailed summary of the species present in each area of the sub-continent. The NER is particularly endemic for *D. immitis* with about an 18% infection rate in Aizwal (Mizoram state) and Guwahati (Assam state) by antigen ELISA test which is specific for *D. immitis* [23]. As the present study employed PCR of blood with general primers for the detection of filariid worms, it was also able to detect species of filariids which are different from *D. immitis*. The detection of the non-pathogenic *A. reconditum* in the NER in our study is important as this is a differential diagnosis for the pathogenic *D. immitis* when seen in blood smears or detected morphologically by Knott’s test. Interestingly, the mildly pathogenic *D. repens* reported from other parts of India [22] was not found in the present study.

Forty-four of the 68 infected dogs (65%) in this study had co-infections with tick-borne pathogens which included *B. gibsoni*, *H. canis* and *A. platys*. The presence of a high rate of co-infections can be attributed to transmission of vectors by the same tick species, high vector intensity and exposure to infected ticks, and increased susceptibility to infection in dogs that are already immune-suppressed by one tick-borne pathogen. In the case of *B. gibsoni* and *H. canis* co-infection, these pathogens are probably transmitted by the same vector tick, *R. sanguineus* (*s.l.*), in the study area; however, while *B. gibsoni* sporozoites are transmitted *via* the tick’s saliva, *H. canis* is transmitted by oral ingestion of the vector tick containing mature oocysts in their hemocoel [14]. Co-infection with *H. canis* in dogs from India was also reported by Abd Rani et al. [9]; nonetheless, co-infection cases in this study were of a considerably lower prevalence and involved *B. vogeli* and *H. canis* in Mumbai (14%) and Delhi (7%), and not *B. gibsoni*. Another study from Kerala in southern India focused on the presence of hemoparasite DNA in dogs and ticks infesting them as detected by multiplex PCR [24]. This study reported the presence of *B. vogeli*, *E. canis* and *B. gibsoni* in *R. sanguineus* (*s.l.*) ticks, while *Haemaphysalis bispinosa* ticks harbored only *B. gibsoni*, thus arousing the suspicion (which needs further experimental substantiation) that this tick species may also serve as a vector of *B. gibsoni*, in addition to *H. longicornis* and *H. hystricis* [19, 20, 24].

The lack of association between the presence of infection with sex and being purebred found in the present study corroborates findings from other studies on dogs in India [9, 25]. *Babesia gibsoni*, the most prevalent CVBD agent found in this study, is a small form *Babesia* species that causes a severe disease in dogs but may also infect dogs sub-clinically. It was initially described from dogs and golden jackals in India by Patton in 1910 [26] and has since been reported to be prevalent in many other parts of the world including eastern Asia, Australia, the Americas and also sporadically in Europe [27]. In addition to being transmitted by ticks, it has also been implicated as being transmitted directly from dog to dog, presumably by bites [28, 29]. *Hepatozoon canis*, the second most prevalent CVBD agent in this study, is a haemogregarnid protozoan that infects canine leukocytes and usually causes sub-clinical to mild infections, but may also induce severe infection [30, 31]. It was also described for the first time in India by James in 1909 [32]. The fact that these two infections were very frequent in the studied dogs and also frequently presented as co-infections, may be associated with their ability to cause sub-clinical infection in the dog, and induce clinical disease occasionally in the presence of immune suppression [27, 33]. The interaction between these infections should be studied further to evaluate if they present together with a more severe disease than when presented as single infection.

Our study was limited by the relatively small number of dogs included, the fact that the clinical signs found in the dogs were not sufficiently detailed, and the absence of information on the identity of ticks present on these animals. Despite these limitations, the findings of the study, carried out in a relatively remote area of India where no molecular studies of hemoparasites in dogs have been previously undertaken, provide important new information in particular about *B. gibsoni* and *H. canis* infections and their co-infection. More studies are required to learn about the risk factors of CVBD in the NER of India, possible interactions between the infecting agents and prevention of these infections.

## Conclusions

A high prevalence of *B. gibsoni* and *H. canis* infection, and their co-infection was found in dogs presented with clinical disease in Mizoram and Tripura states in the southern part of Northeast India. Prevention of CVBD by topical or environmental insecticides and preventive treatment in the case of filarial infection are warranted to decrease the prevalence of these infections. In addition, it is imperative to search for co-infections in dogs with CVBD in India and elsewhere.

## Abbreviations

NER: Northeast Region of India
CVBD: canine vector-borne disease/s
PCR: polymerase chain reaction
EDTA: ethylenediaminetetraacetic acid
NTC: non-template control

## References

[1] Menezes R, Mesquita MM (2008). Incidence of human rabies. World Health..

[2] Sudarshan MK, Mahendra BJ, Madhusudana SN, Ashwath Narayana DH, Rahman A, Rao NSN (2006). An epidemiological study of animal bites in India: results of a WHO sponsored national multi-centric rabies survey. J Commun Dis..

[3] Peleg O, Baneth G, Eyal O, Inbar J, Harrus S (2010). Multiplex real-time qPCR for the detection of *Ehrlichia canis* and *Babesia canis vogeli*. Vet Parasitol..

[4] Rufino CP, Moraes PHG, Reis T, Campos R, Aguiar DCF, McCulloch JA (2013). Detection of *Ehrlichia canis* and *Anaplasma platys* DNA using multiplex PCR. Vector Borne Zoonotic Dis..

[5] Wongkamchai S, Monkong N, Mahannol P, Taweethavonsawat P, Loymak SFS (2013). Rapid detection and identification of *Brugia malayi*, *B. pahangi*, and *Dirofilaria immitis* by high-resolution melting assay. Vector Borne Zoonotic Dis..

[6] Tabar MD, Altet L, Francino O, Sánchez A, Ferrer L, Roura X (2008). Vector-borne infections in cats: molecular study in Barcelona area (Spain). Vet Parasitol..

[7] Almeida AP, Marcili A, Leite RC, Nieri-Bastos FA, Domingues LN, Martins JR (2012). *Coxiella* symbiont in the tick *Ornithodoros rostratus* (Acari: Argasidae). Ticks Tick Borne Dis..

[8] Birkenheuer AJ, Levy MG, Breitschwerdt EB (2003). Development and evaluation of a seminested PCR for detection and differentiation of *Babesia gibsoni* (Asian genotype) and *B. canis* DNA in canine blood samples. J Clin Microbiol..

[9] Abd Rani PAM, Irwin PJ, Coleman GT, Gatne M, Traub RJ (2011). A survey of canine tick-borne diseases in India. Parasit Vectors..

[10] Jain KJ, Lakshmanan B, Syamala K, Praveena JE, Aravindakshan T (2017). High prevalence of small *Babesia* species in canines of Kerala, South India. Vet World..

[11] Vairamuthu S, Ranju RS, Latha BR, Dhivya B, Balachandran C (2014). A six year (2006–2011) retrospective study of hemoprotozoan parasites affecting dogs in Chennai, Tamil Nadu, India. J Parasit Dis..

[12] Patra G, Sahara A, Ghosh S, Behera P, Borthakur SK, Biswas P (2018). Prevalence of tick-borne pathogens in domestic dogs in North-Eastern region of India. Biol Rhythm Res..

[13] Ghosh S, Bansal GC, Gupta SC, Ray D, Khan MQ, Irshad H (2007). Status of tick distribution in Bangladesh, India and Pakistan. Parasitol Res..

[14] Baneth G, Samish M, Alekseev E, Aroch I, Shkap V (2001). Transmission of *Hepatozoon canis* to dogs by naturally-fed or percutaneously-injected *Rhipicephalus sanguineus* ticks. J Parasitol..

[15] Giannelli A, Lia RP, Annoscia G, Buonavoglia C, Lorusso E, Dantas-Torres F (2017). *Rhipicephalus turanicus*, a new vector of *Hepatozoon canis*. Parasitology..

[16] Groves MG, Dennis GL, Amyx HLHD (1975). Transmission of *Ehrlichia canis* to dogs by ticks (*Rhipicephalus sanguineus*). Am J Vet Res..

[17] Zahler M, Schein E, Rinder H, Gothe R (1998). Characteristic genotypes discriminate between *Babesia canis* isolates of differing vector specificity and pathogenicity to dogs. Parasitol Res..

[18] Aktas M, Ozubek S (2018). Molecular evidence for trans-stadial transmission of *Anaplasma platys* by *Rhipicephalus sanguineus sensu lato* under field conditions. Med Vet Entomol..

[19] Higuchi S, Simomura S, Yoshida H, Hoshi F, Kawamura SYY (1991). Development of *Babesia gibsoni* in the hemolymph of the vector tick, *Haemaphysalis longicornis*. J Vet Med Sci..

[20] Jongejan F, Su BL, Yang HJ, Berger L, Bevers J, Liu PC (2018). Molecular evidence for the transovarial passage of *Babesia gibsoni* in *Haemaphysalis hystricis* (Acari: Ixodidae) ticks from Taiwan: a novel vector for canine babesiosis. Parasit Vectors..

[21] Ronghang B, Roy B (2016). Status of tick infections among semi-wild cattle in Arunachal Pradesh, India. Ann Parasitol..

[22] Abd Rani PAM, Irwin PJ, Gatne M, Coleman GT, McInnes LM, Traub RJ (2010). A survey of canine filarial diseases of veterinary and public health significance in India. Parasit Vectors..

[23] Borthakur SK, Deka DK, Islam S, Sarma DK, Sarmah PC (2015). Prevalence and molecular epidemiological data on *Dirofilaria immitis* in dogs from northeastern States of India. Sci World J..

[24] Jain Jose K, Lakshmanan B, Wahlang L, Syamala K, Aravindakshan TV (2018). Molecular evidence of haemoparasites in ixodid ticks of dogs - first report in India. Vet Parasitol Reg Stud Rep..

[25] Singh A, Singh H, Singh NK, Singh ND, Rath SS (2014). Canine babesiosis in northwestern India: molecular detection and assessment of risk factors. Biomed Res Int..

[26] Patton WS (1910). Preliminary report on a new piroplasm (*Piroplasma gibsoni* sp. nov.) found in the blood of the hounds of the Madras hunt and subsequently discovered in the blood of the jackal *Canis aureus*. Bull Soc Pathol Exot..

[27] Solano-Gallego L, Baneth G (2011). Babesiosis in dogs and cats - expanding parasitological and clinical spectra. Vet Parasitol..

[28] Birkenheuer AJ, Correa MT, Levy MG, Breitschwerdt EB (2005). Geographic distribution of babesiosis among dogs in the United States and association with dog bites: 150 cases (2000–2003). J Am Vet Med Assoc..

[29] Jefferies R, Ryan UM, Jardine J, Broughton DK, Robertson ID, Irwin PJ (2007). Blood, bull terriers and babesiosis: further evidence for direct transmission of *Babesia gibsoni* in dogs. Aust Vet J..

[30] Baneth G, Samish M, Shkap V (2007). Life cycle of *Hepatozoon canis* (Apicomplexa: Adeleorina: Hepatozoidae) in the tick *Rhipicephalus sanguineus* and domestic dog (*Canis familiaris*). J Parasitol..

[31] Baneth G, Harmelin A, Presentey B-Z (1995). *Hepatozoon canis* infection in two dogs. J Am Vet Med Assoc..

[32] James SP (1905). On a parasite found in the white corpuscles of the blood of dogs. Sci Mem Off Med Sanit Dep Gov India..

[33] Baneth G (2011). Perspectives on canine and feline hepatozoonosis. Vet Parasitol..

